# Differences Between Expressive Suppression and Cognitive Reappraisal in Opioids and Stimulant Dependent Patients

**DOI:** 10.5812/ijhrba.8514

**Published:** 2013-06-26

**Authors:** Banafsheh Mohajerin, Behrouz Dolatshahi, Abbas Pour Shahbaz, Ali Farhoudian

**Affiliations:** 1 Department of Psychology, University of Social Welfare and Rehabilitation Sciences, Tehran, IR Iran; 2 Department of Psychology, Iranian Research Center for Substance Abuse and Dependence, University of Social Welfare and Rehabilitation Sciences, Tehran, IR Iran

**Keywords:** Methamphetamine, Opioid, Cognitive

## Abstract

**Background:**

Substance use and affective disorders frequently co-occur, but the role of affective dysregulation in addiction is often overlooked. There is evidence shows that substance – dependent individuals have more problems in regulating their emotions.

**Objectives:**

This study compared two commonly used emotional regulation strategies, cognitive reappraisal and suppression, in opioids and methamphetamine dependents.

**Materials and Methods:**

One hundred forty men with substance dependence (70 Opioids, 70 Methamphetamine) were selected by accessible sampling, and they responded to Emotion Regulation Questionnaire (Gross & John) and Clinical drug addiction profile (CDAP) questionnaire. SPSS software was used to analyze the results, and descriptive statistics such as frequency tables and inferential statistics including independent t-test were used.

**Results:**

Opioids and methamphetamine dependent patients differ in reappraisal strategy (P < 0.01). These groups differ not only in reappraisal strategy, but also in the suppression (P < 0.001).

**Conclusion:**

Opioids and methamphetamine dependent individuals used different strategies for regulating their emotions. The key finding was that opioids dependents prefer suppression, and methamphetamine dependents usually use reappraisal for this purpose.

## 1. Background

Substance use disorders are prominent public health concerns. The number of substance abusers in Iran is estimated to be between 1.8 and 3.3 million (1), and it has the highest per capita number of opiate addicts in the world at a rate of 2.8% of Iranians over the age of 15 (2). Amphetamines are the second most commonly used illicit drug type after Cannabis worldwide (3). Methamphetamine use and dependency constitute serious problems not only in Iran but also in a wide area in the world, close to 25 million people worldwide are estimated to use methamphetamine and amphetamine (4), and according to the National Survey of Drug Use and Health, lifetime use of methamphetamine by those 12 and older has ranged from 4.3% in 1999 to a peak of 5.3% in 2002 before falling to 4.9% in 2004 (5). The last report by the Iranian drug control headquarters showed that only 3.6% of substance abusers in Iran used methamphetamine (6). The result of just one study in Iran during 2009-2011, showed that methamphetamine use increased from 6% to about 20% (7). Non official reports estimate that methamphetamine is currently the second or third most widely used illicit substance in Iran (3). Clinical and epidemiological studies have shown a strong association between substance use and affective disorders. Evidences from recent studies show that individuals with affective disorders have high rates comorbidity with substance use disorders. Substance use disorders have also been linked to a range of deficits in the experience and expression of emotion in the absence of affective disorders (8, 9). Anecdotal and empirical evidence both suggest that negative affect and substance dependency are linked together. This association is conceptualized as which individuals who experience greater levels of negative affect are at a higher risk of using coping mechanisms like drugs, food or alcohol to escape from experiencing these emotions (10-14). Theorists and researchers have variously defined the concept of emotion regulation. Most influential definitions were provided by Gross (1998), “process by which individuals influence which emotions they have, when they have them, and how they experience and express these emotions” (15). Thompson defined it as “the extrinsic and intrinsic processes responsible for monitoring, evaluating, and modifying emotional reactions, especially their intensive and temporal features, to accomplish one’s goals’’(16). Individuals use different strategies to alter their emotion; these strategies affect not only their current emotional experience, but also cognitive and interpersonal processes. Emotion regulation is regarded as a crucial factor in well-being and adaptive behavior, and there are different strategies which individuals use for this purpose, but as Garefski (2002), argued some of these strategies are more adaptable than the others (17). Two well-studied regulation strategies are emotional reappraisal and suppression (15), to decrease or increase emotional response tendencies or affective states (18). Suppression reduces emotion-expressive behavior by inhibition during a state of emotional arousal (19). Reappraisal is the reinterpretation of emotionally valence stimuli in unemotional terms (20). It involves generating benign or positive interpretations or perspectives on a stressful situation as a way of reducing distress (21). Reappraisal may be particularly important for psychopathology are beliefs about which emotions are okay to have and which are not (22). Both the reappraisal and suppression of emotional stimuli reduced negative affect. Models of alcohol abuse, suggest that individuals with poor emotion regulation used alcohol to escape from down–regulation of their emotions (23).

## 2. Objectives

The aim of the present study was to examine the differences in emotion regulation between opioid and methamphetamine dependent patients.

## 3. Materials and Methods

The population of this research consists of patients with diagnosed substance dependence as the first axis I diagnosis according to DSM-IV criteria. 140 men (70 opioids, 70 methamphetamines) were selected by accessible sampling from an inpatient substance dependence treatment program of rebirth charity association in Tehran, Iran. The sample age ranged from 18 to 59 years old (Mean ± SD: 32.75 ± 8.35). Ninety one subjects (65%) had used just one type of substance, and 49 ones (35%), had used more than one type during their life. Inclusion criteria for the study were (a), used just one type of substance, opioids (crack, opium or heroin) or methamphetamine for at least 6 months, and (b), their last use was about 14 to 45 days of the date of our study. The exclusion criteria were (a), significant psychiatric disorder (2), and severe brain damage. Data collected between March and August 2012. Information is shown in (Table 1).

**Table 1.** Demographic Characteristics of Opioids and Methamphetamine Dependent Patients.

	Opioids, No. (%)	Methamphetamine, No. (%)
**Marital Status**		
Single	24 (34.3)	33 (47.1)
Married	25 (35.7)	17 (24.3)
Divorced	12 (17.11)	12 (17.1)
Separated	5 (7.1)	7 (10)
Widowed	4 (5.7)	0
**AGE**		
18 - 26	14 (20)	18 (25.7)
27 - 35	35 (50)	35 (50)
36 - 44	10 (14.3)	11 (15.7)
45 - 53	8 (11.4)	5 (7.1)
54 - 62	3 (4.3)	1 (1.4)
**Total**	70	70

Participants were invited to participate in the study after they had been at least 14 days substance free. They used just one of these categories, opioids or methamphetamine for at least six months. Eligible participants had been told that they were under no obligation to participate in the study, although they encourage them to do it. At first, by a face to face interview, participants` demographic information were recorded by means of clinical drug addiction profile (CDAP) by Mokri, Ekhtiari and Farhoudian (2011). This information included age, marital status, education level, family, and risk behaviors history. They described their previous treatments for substance use disorders. Consequently, their psychiatry histories were asked by detail; they were accomplished a five minute rest, afterwards they were asked to fill in the Emotion Regulation Questionnaire (ERQ) by Gross and John with a series of 10 statements. The ERQ assesses typical use of emotion suppression (4 items, e.g., I keep my emotions to myself), and reappraisal (6 items, e.g., When I want to feel less negative emotion, I change the way I am thinking about the situation) in the individual. They sat individually to complete the questionnaire. Then collected data was analyzed by SPSS-16 software. Inferential statistics, independent t-test, bivariate correlation, and Pearson correlation were used to analysis data.

### 3.1. The Emotion Regulation 

Questionnaire (24), is designed to assess individual differences in the habitual use of emotion regulation styles: cognitive reappraisal and suppression. Cognitive reappraisal (When I want to feel more positive emotion, I change the way I am thinking about the situation), and suppression (I keep my emotions to myself). The questionnaire contains 10 items, of which four assess suppression, and the six assess the reappraisal strategy. Participants were asked to rate how they regulate their emotions using a scale from 1 to 7, higher score reflects which strategies individual use more to regulate their emotions. The mean rating across items was computed for each scale to form suppression and reappraisal variables. Gross and John (2003), reported a Cronbach's alpha coefficient reliability value of 0.79 for reappraisal and 0.73 for suppression, and test–retest reliability across three months was 0.69 (24, 25). In the present study The Persian translation of the ERQ was used, which internal coherence estimated by the Cronbach’s alpha coefficient was 0.73 for reappraisal, and 0.54 for suppression.

### 3.2. Clinical Drug Addiction 

Profile (CDAP) by Mokri, Ekhtiari and Farhoudian in collaboration with Ehterami, Farnam, Sefatian, Dolatshahi and Tavajjodi (2011), was used for collecting demographic information. The first part, basic demographic information, includes age, marital status, and education. The second part, drug abuse, profile includes information about the type of drugs used, and also the age, duration, and number of days in which the drug was used in the last month before their participating in the inpatient program. In the third part, treatment history, previous psychiatric treatment for substance use like methadone maintenance treatment, and Naltrexone treatment were asked. Risk behavior profiles include injection, sexual relationship, and criminal history. The fifth part, psychiatric and medical profiles include history of chronic diseases and psychological symptoms like depression, anxiety, and self-harm. The last part, family and social profiles, include occupation, family support and history of substance disorders in their parents.

### 3.3. Data Analysis

For descriptive statistics such as frequency tables and inferential statistics including t- test, bivariate correlation and Pearson correlation, SPSS 16, was used. T-test was used to compare the emotional regulation strategies between mono and poly substance dependents. Bivariate correlation was used to measure the association between emotion regulation strategies and total years of substance used. In this study results with P-value of less than 0.05 were considered significant.

## 4. Result

### 4.1. Group Comparisons

This study was performed on 140 men (70 Opioids, 70 Methamphetamine). For marital status, 57 subjects (40.7%) were single, 42 (30%) married, 24 (17.1%) divorced, 12 (8.6%) separated, and 4 (2.9%) widowed. The average level of education was approximately 11 years (Mean ± SD =11.3 ± 3. 44). Education level of participants was as follows: 23 (16.4%), had maximum primary school diploma, 25 (17.9%) had guidance school up to high school diploma, 92 (65.7%) participants had high- school diploma or higher education. The opioids (opium %15.7, heroin 10.7%, crack 23.6%) dependent group reported using opioids on a mean of 10.08 days last month (SD = 6.73). The methamphetamine dependent reported using methamphetamine on a mean of 9.95 days last month (SD = 5.75). As Table 2 shows, two groups have no significant differences in age, education, and the number of days drug used in the last month before participating in the inpatient program (P < 0.05).

**Table 2.** Comparing Age, Education and the Number of the Day Drug Used in the Last Month Between Opioids and Methamphetamine Dependents (P < 0.05).[Table-fn fn3077]

	No.(Mean ± SD)	df	T-test	Sig. (2. tailed)
**Age**				
Opioids	70 (33.74 ± 8.97)	138	1.40	0.163
Methamphetamine	70 (31.77 ± 7.62)			
**Education**				
Opioids	70 (10.86 ± 3.44)	138	- 0.93	0.35
Methamphetamine	70 (11.40 ± 3.44)			
**Days of drug used in last month**				
Opioids	70 (10.08 ± 6.73)	138	0.121	0.903
Methamphetamine	70 (9.95 ± 5.75)			

P < 0.05

Therefore it seems that opioids and methamphetamine dependent differ in reappraisal strategy. Methamphetamine group showed significantly higher scores on the reappraisal subscale. Therefore it seems that they usually use this strategy to regulate their emotions. These groups were different not only in reappraisal strategy, but also in the suppression. As Table 3 shows, these groups differ in their habitual use of suppression. It seems that the opioids group uses this strategy to regulate their emotions more often.

**Table 3.** Descriptions and Results of T-tests in Comparison Emotional Regulation Strategies Between Opioids and Methamphetamine Dependents

	No. (Mean ± SD)	T-test	df	Sig. (2. tailed)
**Reappraisal**				
Opioids	70 (19.97 ± 5.19)	-4.97	138	0.00
Methamphetamine	70 (25.04 ± 6.75)			
**Suppression**				
Opioids	70 (16.37 ± 4.44)	4.13	138	0.000
Methamphetamine	70 (13.42 ± 3.96)			

To better characterize the difference between opioids and stimulate substance dependent of emotion regulation strategies, we again used t-test base on participations substance history. Descriptions and results of t-tests for these variables are shown in Table 4. No difference was observed on suppression and reappraisal strategies in these groups.

**Table 4.** Descriptions and Results of T-tests for Comparing of Emotional Regulation Strategies Between Mono and Poly Substance Dependents (P < 0.05)

	No. (Mean ± SD)	t	df	Sig. (2. tailed)
**Reappraisal**				
Mono-substance dependent	91 (21.93 ± 5.75)	-1.42	138	0.157
Poly-substance dependent	7049 (23.57 ± 7.71)			
**Suppression**				
Mono-substance dependent	91 (15.19 ± 4.36)	1.08	138	0.282
Poly-substance dependent	49 (14.34 ± 4.59)			

We used bivariate correlation to measure the association between the emotional regulation strategies and total years of substance used. In the second step we tried to find an association between emotion strategies and number of last month’s day substance (number of the days in which drug was used) before they participate in an inpatient program. The result is shown in (Table 5).

**Table 5.** Association (one-tailed Pearson’s r) of Schema Emotion Regulation Strategies with Methamphetamine and Opioids Use

	Suppression Score	Reappraisal Score
**Methamphetamine**		
Total years of substance dependency	- 0.04	0.17
A number of the days methamphetamine used in the last month	0.132	- 0.183
**Opioids**		
Total years of substance dependency	- 0.035	- 0.114
A number of the days opioids used in the last month	0.176	- 0.111

## 5. Discussion

The aim of the present study was to investigate emotion regulation strategies (reappraisal and Suppression) in Opioids and stimulant dependent patients. The result showed that opioids and methamphetamine dependent used different strategies for regulating their emotions. The key finding was that opioids dependents prefer suppression, and methamphetamine dependents usually use reappraisal for this purpose. Depression is prevalent in opiate-dependent patients. The lifetime prevalence rate for major depression was 20% to 50%, and current prevalence rate was in the range of 10% to 20% (26-29). The association between mood disorders and drug use has prompted the hypothesis that patients may often use drugs to blunt or self-medicate their uncomfortable and negative mood state (30, 31). Some researchers stated that the emotional symptoms in depression may be resulted from emotional dysregulation. Suppression is thought to be relevant with emotion regulation strategy in that disorder. Depressed patients not only reported increased suppression of negative affect, but also used suppression for their positive affects (32). Based on some evidences it seems that negative mood states can act as reliable triggers or conditioned stimuli for drug-related responses in opiate dependent patients. Evidence shows that negative induced moods can trigger alcohol desire in some persons (33). Another study showed that smokers with a history of depression have much more difficulty in stopping smoking than the control group (34). Gusse reported that sad mood (Suppression of negative fleeing) was the only exception which does not change during naltrexone treatment. Interventions focusing on reducing depressed mood or anxiety symptoms have been shown to decrease relapse and the severity of alcohol use disorders (35). Moreover, in laboratory paradigms, the induction of negative affect was shown to predict increased urges to drink, and increased expectancies of relief after drinking (12, 36-38). Furthermore, interventions with a strong focus on emotion-regulation skills, such as dialectical behavioral therapy (39), have been shown to reduce substance use (including alcohol) in clients with borderline personality disorder (40, 41). So it seems that opioids dependent patients use drug to suppress their negative affects (35). On the other hand, Methamphetamine causes euphoria, increased energy, and alertness, and enhanced self-confidence. So it seems that methamphetamine dependents are looking for more positive affect. Reappraisal is defined as trying to view situations more positively, as the result showed that they prefer reappraisal to regulate their emotions. These findings are consistent with substantial evidence which suggest that dysregulation of affective processes underlies key aspects of substance use behavior, encompassing vulnerability, early experimentation, as well as the development, and maintenance of substance use disorder.

CBT-based treatments that incorporate mindfulness interventions have shown promise for treating both alcohol-use and drug-use disorders (42-44). These treatments are theorized to enhance the ability to tolerate negative affective states by facilitating a nonjudgmental attitude toward aversive experiences; while, also increasing habituation through formal and informal practices (37, 39). Such treatments include affect regulation training (38, 45) emotion focused therapy (46), and/or the emotion regulation, mindfulness, and distress tolerance modules of dialectical behavioral therapy (47). As opioids dependence prefers suppression to regulation their emotion, therefore therapies focused on emotions seems useful for this population, on the other hand, CBT based therapy would be beneficial for methamphetamine dependence. The result showed that opioids and methamphetamine dependent used different strategies for regulating their emotions. The key finding was that opioids dependents prefer suppression, and methamphetamine dependents usually use reappraisal for this purpose. The study may not be generalized to nonsubstance dependent populations nor people who are under Methadone or Buprenorphine treatment for their substance dependency. The study relies on self-reported emotional regulation estimates, and the nature of the study prevents conclusions about the causal impact of reappraisal/suppression differences in these groups. Future researches might explore how these groups differ with nonsubstance dependent populations, and people use different substance, too.
